# Isolation of B-cells using Miltenyi MACS bead isolation kits

**DOI:** 10.1371/journal.pone.0213832

**Published:** 2019-03-20

**Authors:** Dannielle K. Moore, Bongani Motaung, Nelita du Plessis, Ayanda N. Shabangu, André G. Loxton, SU-IRG Consortium

**Affiliations:** 1 DST-NRF Centre of Excellence for Biomedical Tuberculosis Research; Stellenbosch University, Cape Town, South Africa; 2 South African Medical Research Council Centre for Tuberculosis Research; Stellenbosch University, Cape Town, South Africa; 3 Division of Molecular Biology and Human Genetics, Faculty of Medicine and Health Sciences, Stellenbosch University, Cape Town, South Africa; The Ohio State University, UNITED STATES

## Abstract

This article describes the procedures used to isolate pure B-cell populations from whole blood using various Miltenyi magnetic-activated cell sorting (MACS) bead Isolation kits. Such populations are vital for studies investigating the functional capacity of B-cells, as the presence of other cell types may have indirect effects on B-cell function through cell-cell interactions or by secretion of several soluble molecules. B-cells can be isolated by two main approaches: 1) Negative selection—in which B-cells remain “untouched” in their native state; this is advantageous as it is likely that B-cells remain functionally unaltered by this process. 2) Positive selection–in which B-cells are labelled and actively removed from the sample. We used three Negative B-cell isolation kits as well as the Positive B-cell isolation kit from Miltenyi and compared the purity of each of the resulting B-cells fractions. Contamination of isolated B-cell fractions with platelets was the conclusive finding for all of the isolation techniques tested. These results illustrate the inefficiency of current available MACS B-cell isolation kits to produce pure B-cell populations, from which concrete findings can be made. As such we suggest cell sorting as the preferred method for isolating pure B-cells to be used for downstream functional assays.

## Background

The immune system consists of a collection of cell types responsible for maintaining our health by fighting off infection, eradicating foreign materials and battling disease [1]. B-lymphocytes (B-cells), an immune cell type that forms part of the adaptive immune response, contribute fundamentally to the balance between health and disease. B-cells perform a multitude of effector functions, including antigen presentation, antibody production, cytokine secretion, opsonization, complement activation and immune modulation [2–7]. The activation state of B-cells influences the effect they have on the immune response and ultimately determines whether or not their presence is beneficial or harmful to the host. For example, during autoimmunity regulatory B-cells act to suppress pro-inflammatory, self-reactive T-cell immune responses, thereby protecting the host from self-harm. Whereas, the presence of regulatory B-cells during bacterial infection would result in suppression of antibacterial, protective T-cell immune responses, leading to unsuccessful bacterial containment and poor disease control.

B-cells interact directly with other immunes cells, such as macrophages, T-cells and dendritic cells, through receptor-mediated mechanisms as well as indirectly through the secretion of various molecules. For instance, B-cells present a captured antigen via major histocompatibility complex (MHC) to a T-cell clone within a secondary lymphoid organ resulting in cellular activation, clonal expansion and elicitation of an immune response. This is an example of immune activation. Moreover, B-cells may enhance the function of already activated immune cells through indirect means. For example, antibody secretion by plasma cells (differentiated effector B-cells) enables microbe opsonization which targets foreign material for phagocytosis by circulating macrophages by increasing binding affinity and uptake by endocytosis.

Similarly, B-cell function is influenced by the presence and interaction with other cells types. Several studies have illustrated the necessity of co-stimulation by other cell types via MHC presentation, co-receptor engagement and cytokine encounter for B-cell activation and differentiation [8–17]. An example of receptor-mediated mechanisms that influence B-cell function is the CD40-CD40L interaction that occurs between B-cells and T-cells, required for cellular maturation and survival [10–12]. Additionally, cytokines such as interleukin- 2,4, 6, 21, transforming-growth factor beta (TGF-β) and interferons (IFNs) [9,11,15,17,18] produced by activated immune cells bind to various receptors on the B-cell surface, such as the B-cell receptor, CD21, membrane-bound immunoglobulin and toll-like receptors (TLRs), initiating intracellular signalling pathways that regulate B-cell differentiation and activation. Dysregulation or impaired function of B-cells can result in detrimental consequences for the host; therefore, studies investigating the behavior of B-cells and their contribution to observed immune response are of great importance.

The study of human B-cell populations for functional and/or mechanistic purposes are best performed in the absence of other cell types. The presence of these other cell types may alter B-cell function, either through direct contact or indirectly by the production and secretion of soluble factors like cytokines [15,19,20]. B-cells constitute roughly 10–20% of the total lymphocytes population within whole blood [21–23], as such studies on whole blood, or isolated peripheral blood mononuclear cells (PBMCs) are not suitable for investigating in-depth B-cell specific function. It is thus important to validate isolation procedures that are used to obtain these desirable pure B-cell populations, as they ultimately determine the reliability and accuracy of such studies.

Currently, a range of B-cell isolation kits from various companies are available. Miltenyi B-cell isolation kits are among the most popular to be used for obtaining pure B-cell populations from human [3,24–30] and animal samples [13,29,31,32]. As such, a large proportion of the research currently inferring conclusions regarding B-cell function rely heavily on the efficacy of these kits to isolate B-cells from whole blood. This paper reviews the ability of various B-cell isolation kits available from Miltenyi to isolate pure B-cell populations from human blood, based on the purity of the obtained sample fractions.

## Methods

### Participant recruitment and sample collection/preparation

Ethical approval was obtained from the ethics committee of Stellenbosch University (N16/05/070) and the City of Cape Town City Health. The study was conducted according to the Helsinki Declaration and International Conference of Harmonisation guidelines. For this study, we recruited 27 healthy individuals. On several occasions, varying amounts of peripheral blood was collected in Sodium Heparin tubes and processed as described below. Each blood draw, and subsequent cell isolation procedure, was recorded as a separate event (amounting to a total of 68 cell isolation procedures). Written informed consent was obtained from all study participants.

### Isolation of peripheral blood mononuclear cells (PBMC) from whole blood

In this section, the various protocols amendments used to isolate B-cells from whole blood are described. It should be noted that Miltenyi isolation kits used to obtain enriched pure B populations required a pre-isolation of PBMCs from whole blood. As such the protocol for PBMC isolation refers to the initial processing of blood samples, before the use of either of the Miltenyi isolation kits.

#### Isolation of mononuclear cells from peripheral blood using the Ficoll-density gradient method

Human whole peripheral blood was collected in sodium heparin tubes and processed within two hours of blood draw. PBMCs were isolated using the Ficoll-density gradient method. Following this, PBMCs underwent MACS bead isolation. For a more detailed description regarding PBMC isolation refer to dx.doi.org/10.17504/protocols.io.yf2ftqe [PROTOCOL DOI]

#### Additions and Alterations to PBMC isolation protocol

**A. Addition of platelet wash step to PBMC isolation procedure.** This was done to decrease the debris/platelet population found in the PBMC sample. Alteration of the speed at which the isolated PBMC’s were washed was done in an attempt to prevent pelleting and retention of the platelets during the washing steps. It is assumed that the degree of platelet contamination within the PBMC fraction is likely to affect the purity of the isolated B-cell fraction, and thus should be limited. The alteration steps listed replace step 8 in the PBMC isolation method. Three different approaches were used, each tested in a separate set of experiments multiple times by various lab technicians:

- Wash the PBMCs twice in 50mL of PBS, centrifuge at 300xg for 10 min at room temperature
- Wash the PBMCs twice in 50mL of PBS, centrifuge at 200xg for 10 min at room temperature
- Wash the PBMCs twice in 50mL of PBS, centrifuge at 120xg for 15 min at room temperature

### Isolation of B-cells from mononuclear cells using the Miltenyi cell isolation kits

Miltenyi isolation kits are based on a simple process, in which biotin conjugated antibody-labelled mononuclear cells are separated from unlabeled cells via a column in the presence of a magnetic field. During negative selection, the cell type of interest (in this case B-cells) remains unlabeled/“untouched” and in its native state, while the remaining unwanted mononuclear cells (in this case T-cells, NK cells and monocytes) are targeted via biotin labelled-antibodies specific for cell surface receptor(s) of those cell types. During positive selection, the cell type of interest (in this case B-cells) are specifically targeted, labelled and actively magnetically removed from the sample by retention in the column in the presence of a magnetic field. These cells are then retrieved by plunging the column in the absence of a magnetic field.

The efficiency of various Miltenyi isolation kits were tested. Specifically, 3 negative B-cell isolation kits, 1 positive B-cell isolation kit and 1 T-cell isolation kit were examined (see list below). All kits were operated according to the manufacturer’s instructions. For a more detailed description regarding this process refer to dx.doi.org/10.17504/protocols.io.yfzftp6 [PROTOCOL DOI]

#### List of Miltenyi isolation kits examined

- Isolation of B-cells from mononuclear cells by negative selection using the Miltenyi B-cell Isolation kit II (For detailed description of kit components see Datafile A in S1 File)
- Isolation of B-cells from mononuclear cells by negative selection using the Miltenyi Naïve B-cell Isolation kit (For detailed description of kit components see Datafile D in S1 File)
- Isolation of B-cells from mononuclear cells by negative selection using the Miltenyi CD43 Microbeads kit (For detailed description of kit components see Datafile E in S1 File)
- Isolation of B-cells from mononuclear cells by positive selection using the Miltenyi CD19 positive Isolation kit (For detailed description of kit components see Datafile F in S1 File)

#### Additions and Alterations to Miltenyi B-cell isolation kit II original protocol

**A. Addition of Miltenyi dead cell removal kit to isolation protocol.** The addition of this isolation kit was done in attempt to decrease the “cell debris/platelet” population found within isolated B-cell fractions. This kit was used according to the manufacturer’s recommendations (For detailed description of kit components see Datafile B in S1 File) and integrated into the isolation procedure using three different approaches:

- Perform dead cell removal procedure before B-cells isolation procedure
- Perform dead cell removal procedure simultaneously to B-cell isolation (Addition of dead cell staining buffer together with negative isolation kit buffers–MACS buffer volumes altered to maintain stipulated staining volume of B-cell isolation kit II)
- Perform dead cell removal procedure after B-cells isolation procedure

**B. Addition of Miltenyi CD61 platelet removal kit to isolation protocol.** The addition of this isolation kit was done in attempt to decrease the “cell debris/platelet” population found within isolated B-cell fractions. This kit was used according to the manufacturer’s recommendations (For detailed description of kit components see Datafile C in S1 File) and integrated into the isolation procedure described in section 2.3.1 was done using three different approaches:

- Perform CD61 platelet removal procedure before B-cells isolation procedure
- Perform CD61 platelet removal procedure simultaneously to B-cell isolation (Addition of CD61 staining buffers together with negative isolation kit buffers—MACS buffer volumes altered to maintain stipulated staining volume of B-cell isolation kit II)
- Perform CD61 platelet removal procedure after B-cells isolation procedure

### Fluorescent-activated cell sorting (FACS) of isolated B-cell from samples pre-processed using the Miltenyi B-cell isolation kit II

Lymphocytes were FACS sorted from biological samples, that had already undergone MACS isolation sample using the B-cell isolation kit II, based on their forward-scatter area (FSC) and side-scatter area (SSC) properties. In theory, B-cells should account for the majority (above 90%) of the cells within the lymphocyte population. Therefore, this additional separation process should remove particles within the sample that do not form part of the lymphocyte population, essentially purifying the sample. Sorting based on size and cellular complexity rather than fluorescence was done to limit the level of manipulation and processing that the cells were exposed to, as this may have negative effects on downstream processes as previously mentioned. Prior to sorting the cell suspensions were filtered through 0.35μm filter to remove any cell aggregates that could block the fluidics lines within the FACS instrument. Samples underwent two sorting steps:

- An enrichment sort—to remove excessive debris without compromising the cell numbers by sorting all cells of interest, and
- A pure sort—to completely eliminate cell debris while sacrificing cell yield by only sorting the cells of interest that were not flanked by contaminating cells.

### Immunofluorescence staining and flow cytometric analysis of various cell fractions to determine the purity of isolated samples

Sample purity is of vital importance; this purity check validates the isolation technique as sufficient in isolating the cell type of interest and that conclusions drawn from downstream experiments are conclusive based on the measured cellular responses of the isolated cells and not by the presence/influence of other cell types.

The isolated fraction was stained with anti-human mAb specific for CD19 (to determine the proportion of isolated B-cells in the lymphocyte population i.e “B-cell purity”) and anti-human mAb specific for CD36 (to determine the proportion of platelets making up the cell debris population i.e. “Platelet Contamination”). The differentiation of cell debris from lymphocytes was determined using FSC and SSC, a due to the fact that cell debris, dead cells and platelets are of a smaller size and less cellular complexity compared to lymphocytes. The gating strategy used to evaluate sample purity of isolated B-cells (see Figure A in S1 File and Figure B in S1 File) and T-cells (Figure C in S1 File) is displayed in Supplementary data. The resulting data was analyzed using FlowJo v10 software (Oregon, USA).

### Statistical analysis

Data analysis of the flow cytometry plots was done using FlowJo V10 (Treestar, USA) and the resulting stats analyzed using Prism 7 Software (San Diego, CA). Statistical differences between groups was calculated using a non-parametric Kruskal-Wallis test with a Dunn’s multiple comparisons test. Alternatively, when applicable a multiple non-parametric unpaired student t-test was used to calculate statistical differences between groups for a list of independent variables. Linear regression analysis was performed using Prism 7 Software. A two-way step-up Benjamini, Krieger and Yekutieli False Discovery rate (FDR) approach, with an FDR of 1%, was used to correct for multiple testing. Statistical significance is indicated by an asterisk, in which the p < 0.05 (*), p<. 0.01 (**), p<0.001(***) and p<0.0001(****) or by letters in which data points with different letters indicated statistical differences.

## Results and discussion

**Fig 1:**
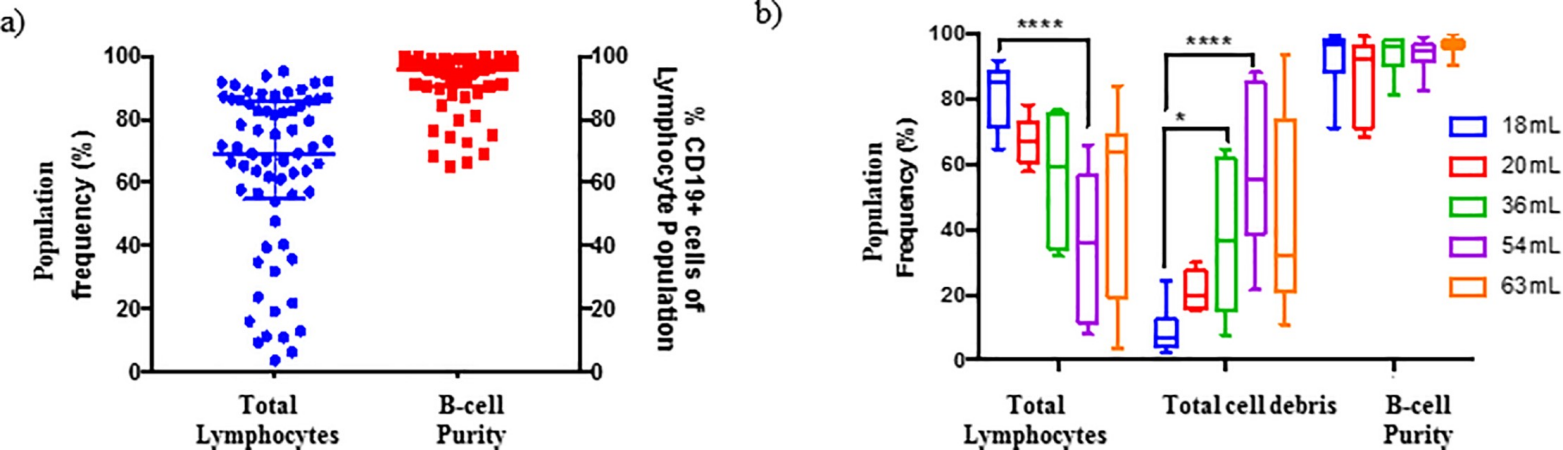
Analysis of isolated B-cell sample purity obtained using commercially available MACS B-cell isolation kit II (n = 68). (a) Sample purity following negative MACS bead isolation using B-cell Isolation Kit II only. The left axis illustrates total lymphocytes as a percentage of all cellular content within the isolated sample, while the right axis illustrates B-cell purity as a percentage of the lymphocyte population (b) Effect of blood volume on effectiveness of MACS B-cell Isolation Kit II. Statistical differences between blood volumes was calculated using a non-parametric Kruskal-Wallis test with a Dunn’s multiple comparisons test. A two-way step-up Benjamini, Krieger and Yekutieli False Discovery rate (FDR) approach, with a FDR of 1%, was used to correct for multiple testing. Statistical significance is indicated by an asterisk, in which the p < 0.05 (*), p<. 0.01 (**), p<0.001(***) and p<0.0001(****).

The purpose of cell isolation methods is to obtain a pure cell population of interest, that can be used to investigate cell structure and function without the influence of other cell types. As such, the presence of contaminants in an isolated sample are undesirable and defeat the objective of isolation procedures. The methods described in this paper using various Miltenyi B-cell isolation kits result in such a phenomenon. The desired purity of a cell-type within an isolated sample equates to 90% or more of the total cell population. Key factors used to assess the purity of isolated samples within this study included: 1) lymphocyte population frequency—the proportion (%) of lymphocytes within the sample, 2) debris population frequency—the proportion (%) of cell debris within the sample, 3) Platelet frequency–the proportion (%) of CD36^+^ within the debris population and 4) B-cell purity–the proportion (%) of CD19^+^ within the lymphocyte population. It should be highlighted that the acceptable > 90% isolated cell purity stated in the data sheet of the tested isolation kits is only achieved when examining the cellular content of the lymphocyte population, while excluding all cell debris from analysis (Fig 1A). This type of analysis is termed “gated purity” and overlooks the composition of the isolated sample as a whole. This isolation method is sufficient for studies only focusing on cell surface receptor expression analysis but not functional downstream experiments, due to the presence of cellular contaminants within the final sample fraction. Thus “gated purity” should not be the only purity of concern when assessing the efficiency of isolation procedures. In almost all instances, with the exception of the MACS CD19 positive isolation kit, platelets were found to be a large contaminant within the isolated samples, making up a large portion of the total cell content within the isolated fraction (Table 1). Platelets were identified using flow cytometry based on their size, cellular complexity and expression of the cell surface marker CD36 [33,34]. This large variation of platelet contamination may be as a result of individual sample variation in the number of platelets per liter of human peripheral blood–the reference range has been reported as 150–450 x 10^9^ per liter [35–37].

**Table 1:** Summary of B-cell isolation kits tested. This table includes the various parameters used to evaluate the efficiency of each of the Miltenyi B-cell isolation kits tested. The statistical comparison between the various parameters listed above is graphically illustrated in Fig 3.1.

	Total Lymphocytes (% of sample cellular content)		Total cell debris (% of sample cellular content)		Platelet population (% of Debris population)		B-cell Purity (%CD19^+^ cells of lymphocyte population)		Blood Volume (mL)	N
	Median	Range	Median	Range	Median	Range	Median	Range	Range	
Naive B kit	11,20	-	87,10	-	94,40	-	96,90	-	36	1
CD43+ kit	11,30	-	85,40	-	83,30	-	61,90	-	36	1
CD19+ kit	52,00	20,30–62,20	40,70	30,00–71,40	0,39	0,26–1,82	92,40	91,10–96,20	20	3
B-cell Isolation Kit II + CD61 removal kit	70,90	65,80–73,40	9,38	7,61–18,60	54,90	52,20–69,90	99,00	65,70–99,30	18	3
B-cell Isolation Kit II + Dead cell removal kit	17,60	16,30–63,30	80,60	34,50–82,80	90,70	72,00–92,00	98,70	98,30–99,50	18	3
B-cell Isolation Kit II	69,15	3,63–95,20	17,85	1,48–93,60	86,55	0,014–99,70	95,75	64,90–99,90	18–63	68

**Fig 2:**
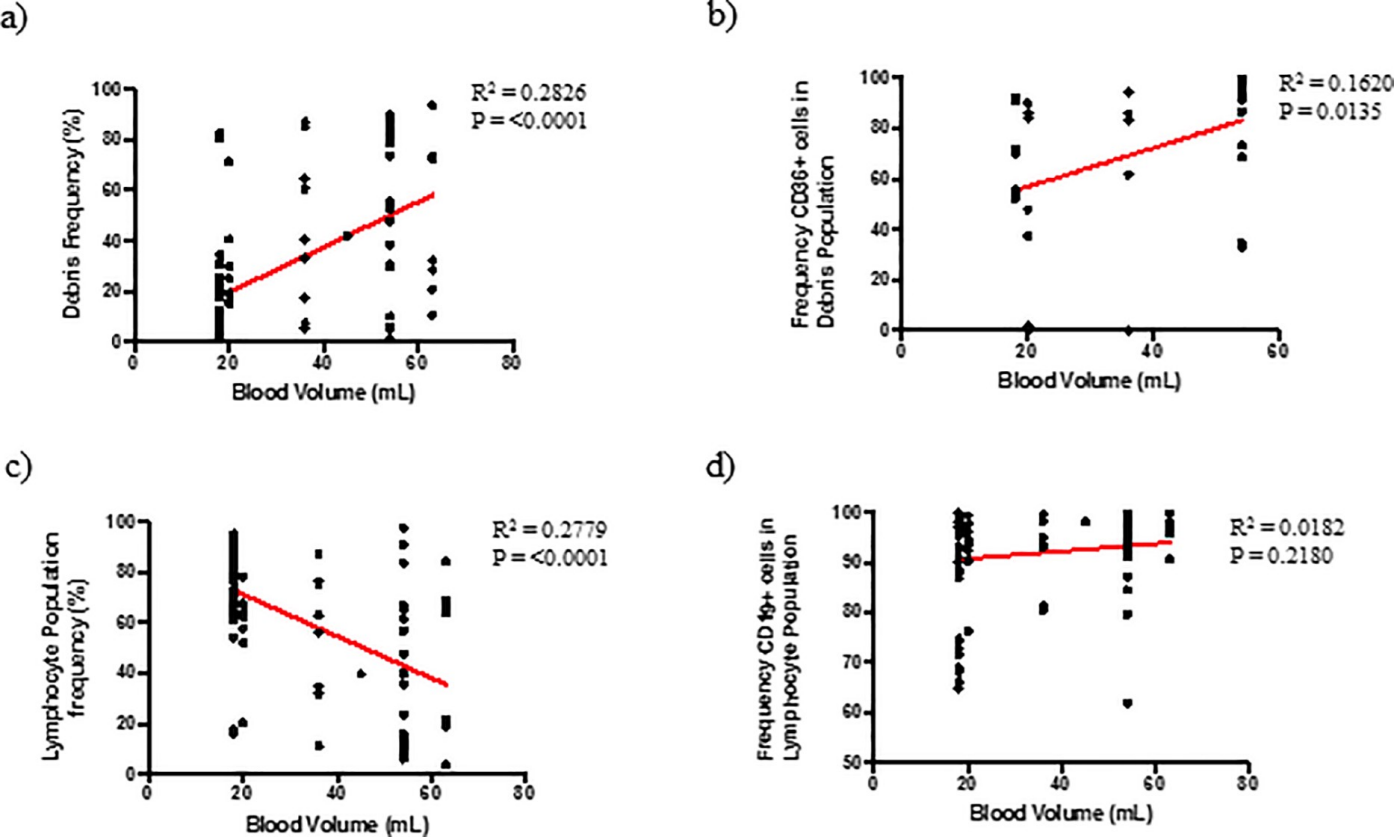
Relationship between B-cell isolation quality and sample volume for all B-cell isolation kits tested (n = 79). (a) Linear regression between blood volume and frequency of debris population (b) Linear regression between blood volume and total lymphocyte population (c) Linear regression between blood volume and frequency of CD36+ platelets within debris population (d) Linear regression between blood volume and frequency of CD19+ cells in lymphocyte population.

Consequently, the volume of peripheral blood used for the isolation procedure, and in turn the number of platelets comprised within the whole blood sample, had a significant effect on the obtained sample purity (Fig 1B). A significant difference for each of the factors used to assess sample purity following cell isolation was observed when assessing the effect of starting blood volume on procedure efficiency. Upon further analysis it was found that a positive relationship exists between whole blood volume and the frequency of the debris population (Fig 2A), as well as platelet contamination (Fig 2B). Conversely, a negative relationship exists between whole blood volume and the frequency of the lymphocyte population (Fig 2C). Notably, the frequency of CD19^+^ cells within the lymphocyte population remained unaffected by whole blood volume (Fig 2D).

As previously stated, significantly less platelet contamination was observed in samples isolated with the MACS CD19 positive isolation kit, when compared to the negative B-cell isolation kit II (Table 1). However, the obtained isolated sample comprised of a large proportion of ‘cell debris’ (range 30–70%). Characteristically, dead cells are identified in flow cytometry by changes in their light scatter; generally they exhibit decreased FSC and increased in SSC properties [38–41], hence are suspected to make up this debris population. During positive selection surface receptors on the cell type of interest are targeted for labeling. Theoretically, positive selection should result in highly pure cell populations to be obtained, as all non-labelled cells (including cell debris) should simple elute from the column leaving only the labeled cell type of interest. However, non-specific binding may occur resulting in unwanted cell types contaminating the final sample. Dead cells have been known to bind non-specifically to antibodies [40,41] and could thus be the major source of the observed contamination. Nevertheless, further investigation is required in order to determine the source of this ‘contamination’. Ideally, negative isolation would be the method of choice as the cell type of interest surface receptors remain unbound and thus functionally unaltered by this process, where as positive selection introduces the risk of possible cell activation and ultimately functional alteration of the cell, which may complicate downstream processes. Possible downstream processes include but is not limited to investigating cellular activation following exposure to a particular molecule/drug. This *ex vivo* manipulation could result in altered B-cell function to either enhance or diminish the measured B-cell response resulting in artefactual observations.

Of the three negative B-cell isolation kits investigated, one of the kits, namely the CD43 microbead isolation kit, did not contain any anti-biotin CD36^+^ monoclonal antibody (mAb), which would explain the large degree of platelet contamination within these samples (Table 1, Figure B in S1 File). However, a large population of CD36^+^ platelets were identified within isolated samples that underwent processing using either of the other two negative B-cell isolation kits that contain an anti-biotin CD36 mAb (Table 1), namely the B-cell isolation kit II (Figure D in S1 File) or the Naïve B-cell isolation kit (Figure B in S1 File), for effective removal of CD36 positive cells (including platelets). A possible reason for the observed phenomenon is inadequate concentration of this cell surface receptor mAb within the antibody cocktail solution, thereby influencing the obtained sample purity. This supports the observed effects of whole blood volume of the overall effectiveness of the isolation process, in which an increase in the volume of starting sample, hence platelet numbers, resulting in decreased efficiency in removal of CD36+ platelets. Due to the large individual variability of platelet counts per participant, it is difficult to determine how much anti-biotin CD36 mAb is adequate.

**Fig 3:**
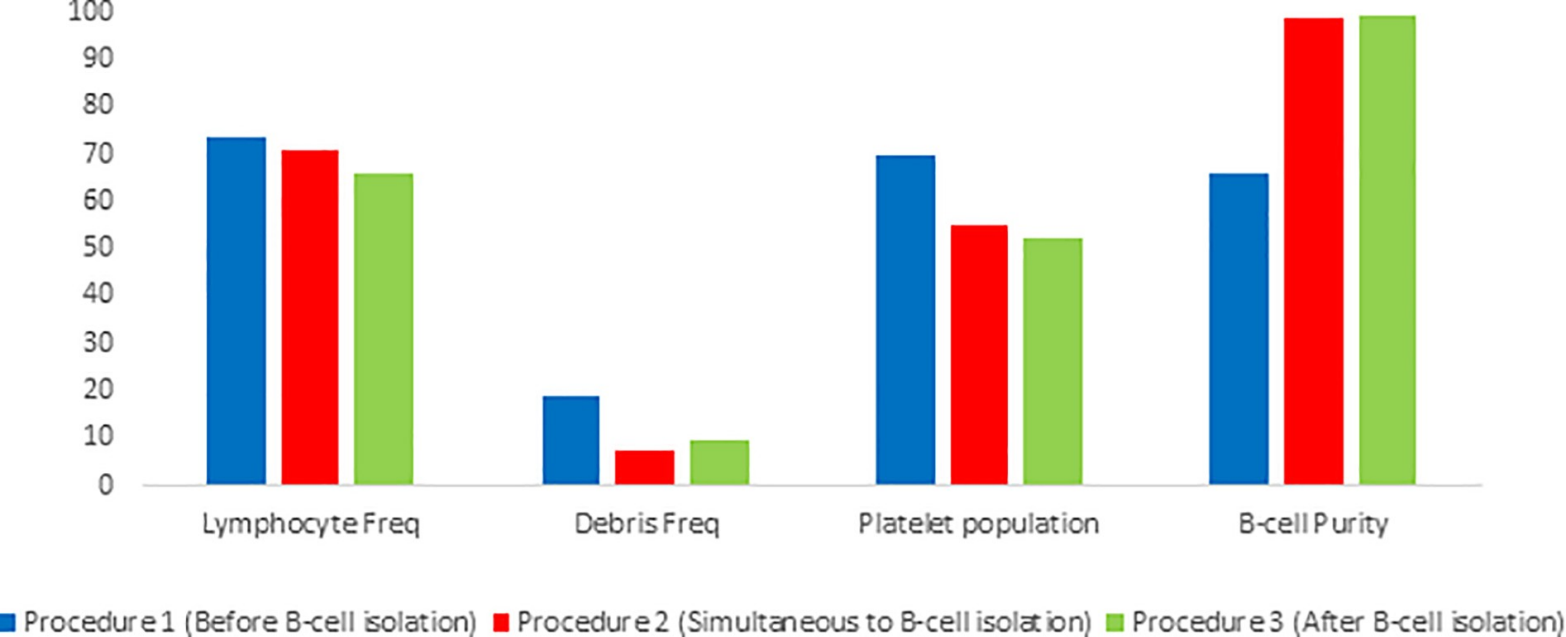
Analysis of isolated B-cell sample purity obtained using the MACS B-cell isolation kit II with the addition of a CD61 platelet removal kit. The implementation of the CD63 platelet removal kit was done in three different ways, namely before isolation with MACS B-cell isolation kit II (Procedure 1, n = 1), simultaneously with MACS B-cell isolation kit II (Procedure 2, n = 1) and after isolation with MACS B-cell isolation kit II (Procedure 3, n = 1).

**Fig 4:**
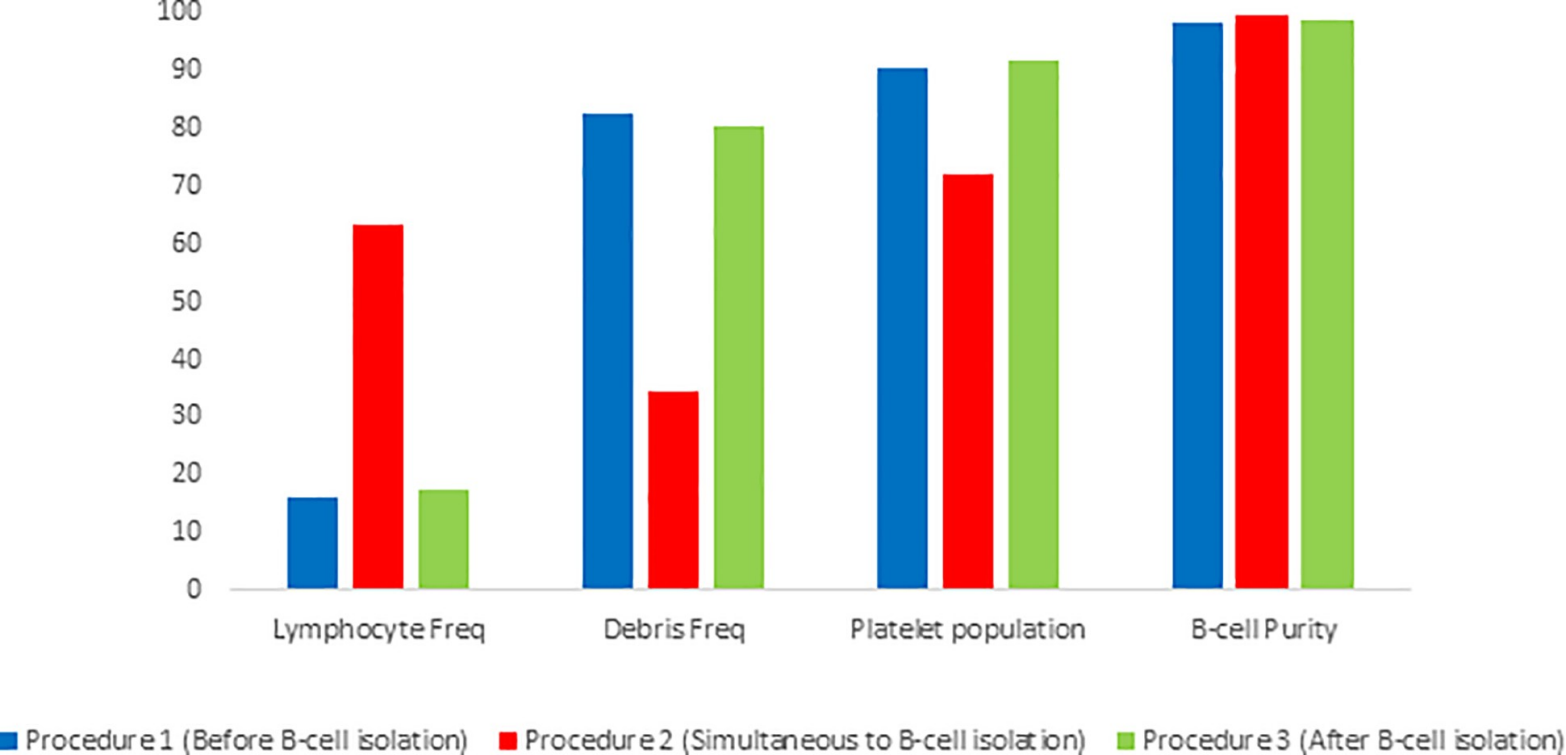
Analysis of isolated B-cell sample purity obtained using the MACS B-cell isolation kit II with the addition of the dead cell removal kit. The implementation of the dead cell removal kit was done in three different ways, namely before isolation with MACS B-cell isolation kit II (Procedure 1, n = 1), simultaneously with MACS B-cell isolation kit II (Procedure 2, n = 1) and after isolation with MACS B-cell isolation kit II (Procedure 3, n = 1).

Various steps were implemented into the isolation procedure utilizing the MACS negative B-cell isolation kit II, in an attempt to decrease the platelet contamination and ‘cell debris’ population found within isolated samples. These modifications including the addition of a MACS CD61 platelet removal kit to determine whether or not this would improve the resulting sample purity (Figure A in S1 File). While a general improvement in the obtained sample quality was observed as seen by a reduction in the frequency of the debris population within the isolated sample, these differences were not statistically significant from either of the other isolation methods (Table 1). It should be noted that when comparing the effectiveness of the addition of the MACS CD61 microbead kit to other isolation methods, the results from procedure I, II and III were pooled. When subjectively relating each of the implementation methods separately, no methods was perceived to be superior (Fig 3). Furthermore, the addition of a MACS dead cell removal kit was implemented to determine whether this would improve sample quality in cases where platelet contamination within the “cell debris” population was infrequent (Figure A in S1 File). No improvement in the resulting sample quality was observed (Table 1). It should be noted that when comparing the effectiveness of the addition of the MACS dead cell removal kit to other isolation methods, the results from procedure I, II and III were pooled. Notable, when subjectively relating each of the implementation methods separately, procedure II was perceived to yield sample purity of a superior quality (Fig 4). As such, further investigation utilizing method II should be performed in order to better evaluate the usefulness of this kit in improving B-cell isolation.

Additionally, the effectiveness of this MACS dead cell removal kit was not investigated in combination with the MACS CD19 positive isolation kit, in which dead cells are the suspected cause of contamination within isolated samples (Figure B in S1 File). Accordingly, additional investigation is required before a conclusive decision can be made with regards to the efficiency of the modification in refining the isolation process.

**Fig 5:**
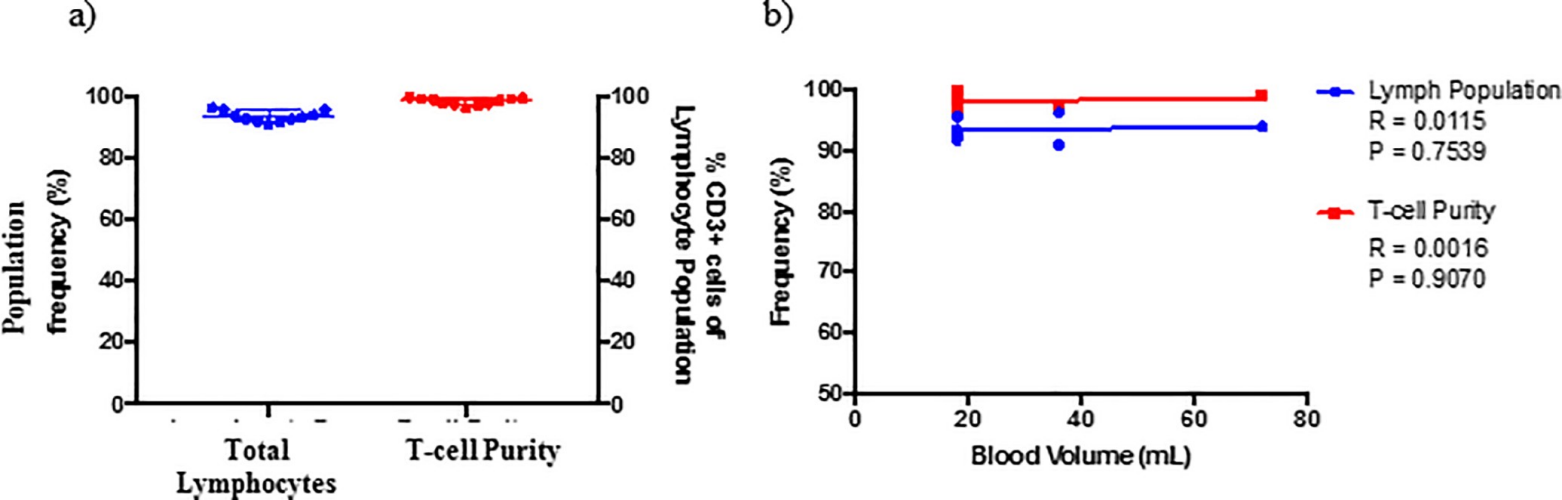
Analysis of isolated T cell sample purity obtained using the MACS Pan T cell isolation kit (n = 11). (a) Sample purity following negative MACS bead isolation. The left axis illustrates total lymphocytes as a percentage of all cellular content within the isolated sample, while the right axis illustrates T-cell purity as a percentage of the lymphocyte population. As illustrated, platelet/cell debris was successfully removed, and a pure T cell population obtained, as shown by CD3^+^ cells (b) Linear regression between blood volume and efficiency of MACS Pan T-cell isolation kit with reference to frequency of lymphocyte population and frequency of CD3^+^ cells within lymphocyte population (T-cell Purity).

Interestingly, when performing T cell isolations using the MACS Pan T cell negative isolation kit available from Miltenyi, limited debris/platelet contamination within the resulting isolated fraction was observed (Fig 5A). Additionally, sample volume was observed to have no confounding effect on the resulting sample purity (Fig 5B). These results thereby illustrate that our PBMC isolation technique and MACS isolation methods are up to standard and are not the cause of this recurring contamination issue. Instead, these results emphasize the inefficiency of current B-cell isolation kits available from Miltenyi to produce pure B-cell populations from which concrete findings can be made. Given that it is standard practice for immunology research to be conducted on immune cells in isolation *ex vivo* [3,13,24–29,29–32], it is crucial that any external influences, such as the presence of contaminating cells within an isolated sample, be acknowledged as these factors may contribute to the measured physiological responses, resulting in artefactual observations. It is thus proposed that the Miltenyi B-cell isolation kits are suboptimal and need further optimization in order to achieve the desired cell isolation results for which these kits are intended. The presence of platelets and cell debris within isolated cell samples to be used for downstream functional assays is a significant drawback—as these “contaminants” may influence the function of cells under investigation. Platelets and cell debris contribute to the microenvironment, that is the *in vitro* setting, in which these cells are investigate as they secrete various substances such as cytokines (in the case of platelets) or cellular content (in the case of dead cells) known to have an effect on the activation and function of surrounding immune cells [42,43]. Thus, the observed immune response of investigated cells may not be identical to those where pure sample isolates were used as the cellular function may have been altered by the substances released from these contaminating cells. Evidence has illustrated that platelets have the capacity to modulate B and T cell function—and potentially drive their operation [18]. One such way in which platelets may achieve this is through the expression of mRNA found within their cytoplasm [44–46]. The resulting proteins include a variety of molecules known to influence cell function, including cell surface receptors involved in cellular activation and various cytokines [18,42,43]. These findings are of huge concern, as several studies inferring B-cell function downstream utilizing these kits to obtain “pure” B-cell populations, have not acknowledged the presence of these platelets within their isolated samples. Therefore, any conclusions made from isolated cell studies that utilized these B-cell isolation kits, need to consider the presence of these platelets within the sample, and note the limitations when reporting their findings contributing to B-cell function.

**Fig 6:**
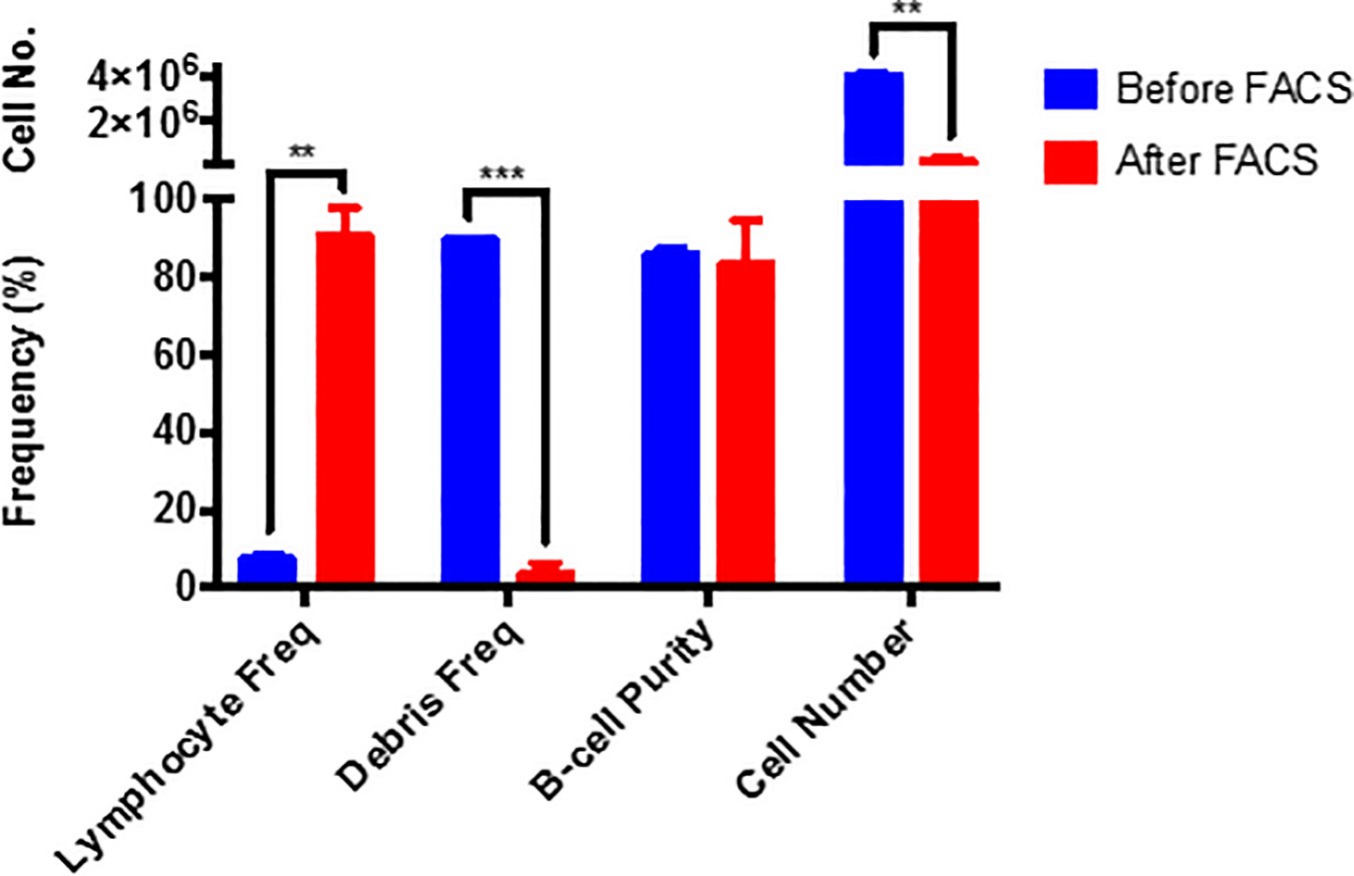
Analysis of isolated B-cell sample purity obtained using the MACS B-cell isolation kit II, followed by cell sorting based on FSC and SSC (n = 2). Statistical differences between sorting conditions was calculated using multiple non-parametric unpaired student t-tests. A two-way step-up Benjamini, Krieger and Yekutieli False Discovery rate (FDR) approach, with a FDR of 1%, was used to correct for multiple testing. Statistical significance is indicated by an asterisk, in which the p < 0.05 (*), p<. 0.01 (**) and p<0.001(***).

Lastly, the addition of a fluorescent-activated cell sorting (FACS) step, following B-cell isolation with the Miltenyi B-cell isolation kit II, was investigated to determine whether this could improve sample purity. The resulting sample fraction was found to have significantly improved sample purity, however cell number was compromised using this method (Fig 6). The gating strategy used to evaluate sample purity of FACS B-cells is displayed in Figure D in S1 File. Based on the findings of this paper, the addition of FACS sorting to this isolation procedure for obtaining pure B-cell populations is recommended, as this technique was found to significantly reduce the platelet contamination within the isolated sample fractions. Consequently, the tested B-cell isolation kits would better serve as a pre-enrichment step prior to cell sorting rather than an isolation technique alone. This is highly beneficial in comparison to traditional cell sorting as it allows for the isolation of naïve B-cells, whereas B-cells sorted directly from whole blood or PBMC samples requires labeling of the cells of interest with a fluorescent tag for removal from cell suspension. This is disadvantageous as it may result in cell activation through engagement of the B-cell receptor (CD19) resulting in altered immune function, as well as the increased amount of time needed to sort the B-cells out of these dense cell populations, resulting in excessive costs. As such, the use of the commercially available MACS isolation kits is not discouraged but rather recommended as an additional pre-step to cell sorting procedures.

Various FACS sorting platforms exist (eg. BD, SONY, Beckman Coulter) including the MACSQuant-Tyto (Miltenyi Germany) and use of the services offered should be to achieve the desired sample purity. However, in terms of the research cost, processing time and the volume of blood required to obtain sufficient B-cell numbers for functional assays downstream, this method may not be feasible for most laboratories. A possible improvement to reduce the loss in cell number could be to label the sample with a CD36 mAb and actively sort the platelets within the debris population, using the pure sort method, based on their FSC and SSC properties as well as fluorescence; followed by a pure sort of the lymphocyte population based on FSC and SSC, with less abort rates as the sample will comprise of significantly less cell debris. Importantly, B-cell isolation kits from additional companies was not investigated within this study. Therefore, the results depicted within this paper do not dissuade the efficacy of other B-cell isolation kits. However, it would be advisable to do a comparative study to determine the efficacy of the kits not tested.

## Supporting information

S1 File **Bcell Isolation kit II (Datafile A). Dead Cell Removal Kit (Datafile B). CD61 Microbeads (Datafile C). Naïve B cell Isolation Kit II (Datafile D). CD43 Microbeads (Datafile E). CD19 Microbeads (Datafile F). Flow cytometric analysis of isolated B cell sample purity obtained using the B cell isolation kit II.** (a) Using the normal PBMC method (n = 16) (b) Using the altered platelet wash method (n = 4) c) Addition of dead cell removal kit (n = 3) (d) Addition of CD61 platelet removal kit (n = 3) **(Figure A). Flow cytometric analysis of isolated B cell sample purity obtained using the naive isolation kits.** (a) Naïve B cell Isolation kit (n = 1) (b) CD43 microbeads kit (n = 1) (c) CD19 positive Isolation kit (n = 3). These sample were not stained with CD36 mAb and thus the proportion of platelets that make up the ‘Debris’ population cannot be confirmed **(Figure B). Flow cytometric analysis of isolated T cell sample purity obtained using the Pan T cell isolation kit.** Sample purity following negative MACS bead isolation in which platelet/cell debris was successfully removed, as shown in SSC-A vs FSC-A, and a pure T cell population obtained, as shown by CD3+ cells (n = 11) **(Figure C). Flow cytometric analysis of isolated B cell sample purity obtained using the B cell isolation kit II, followed by cell sorting based on FSC and SSC.** a) Sample purity of MACS bead isolated B cell sample b) Sample purity of MACS bead isolated B cell sample followed by two cell sorting steps, resulting in successfully removal of undesirable platelet contamination (n = 2) **(Figure D)**.
(DOCX)
