# Supplementary material for: Isolation of B-cells using Miltenyi MACS bead isolation kits
Source: PLoS One. 2019 Mar 20;14(3):e0213832. doi: 10.1371/journal.pone.0213832 (PMC6426237; doi:10.1371/journal.pone.0213832)

**Supplementary Information**

S1 Datafile A Cell Isolation kit II


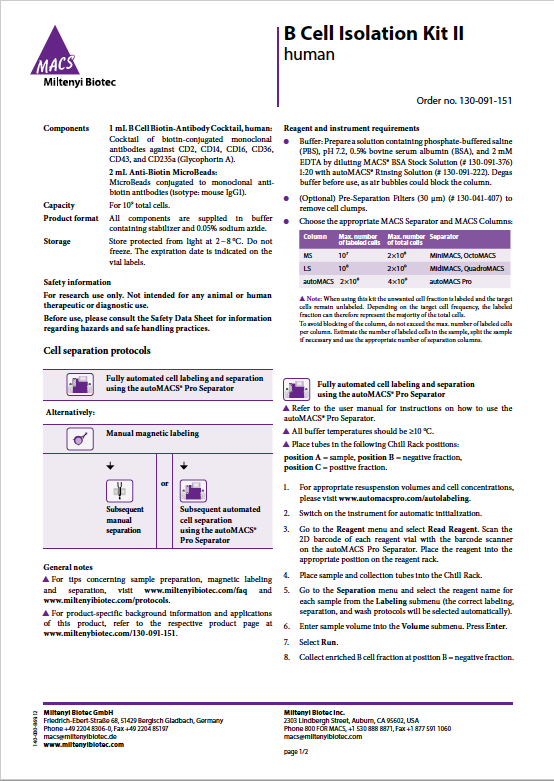


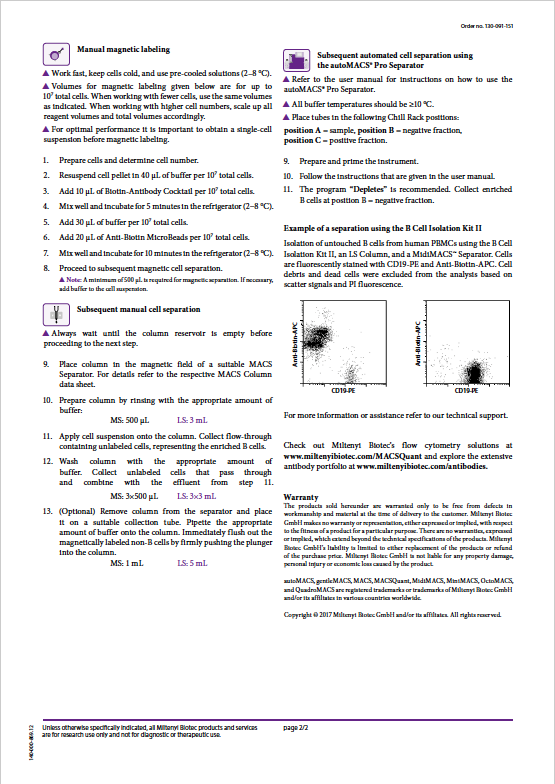


S1 Datafile B Dead Cell Removal Kit


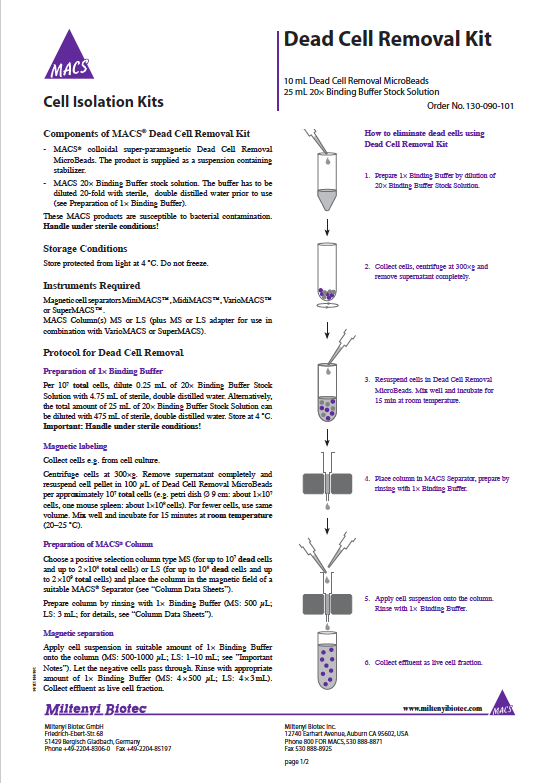


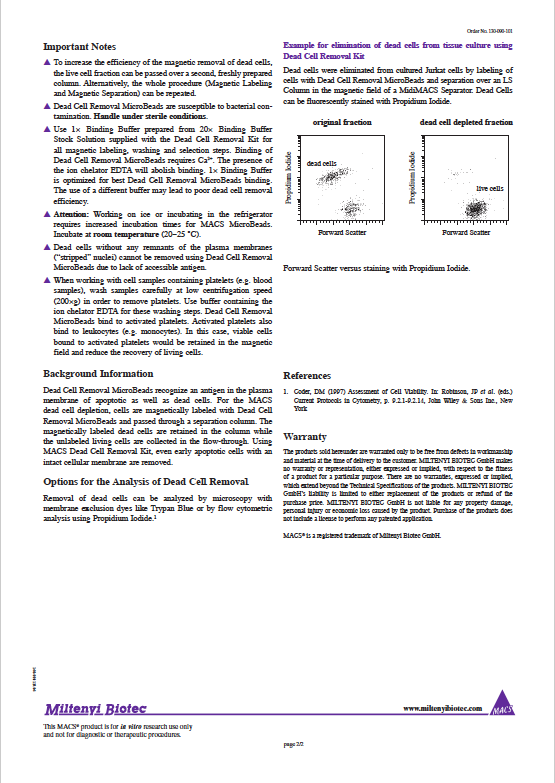


S1 Datafile C CD61 Microbeads


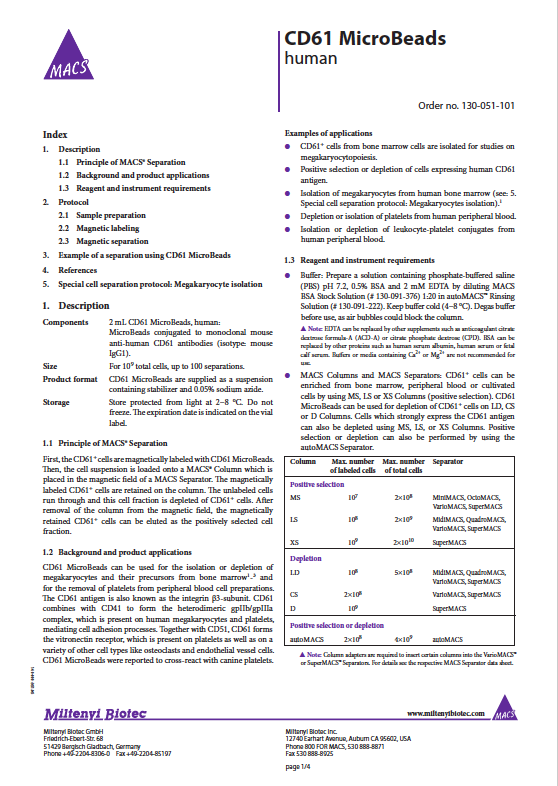


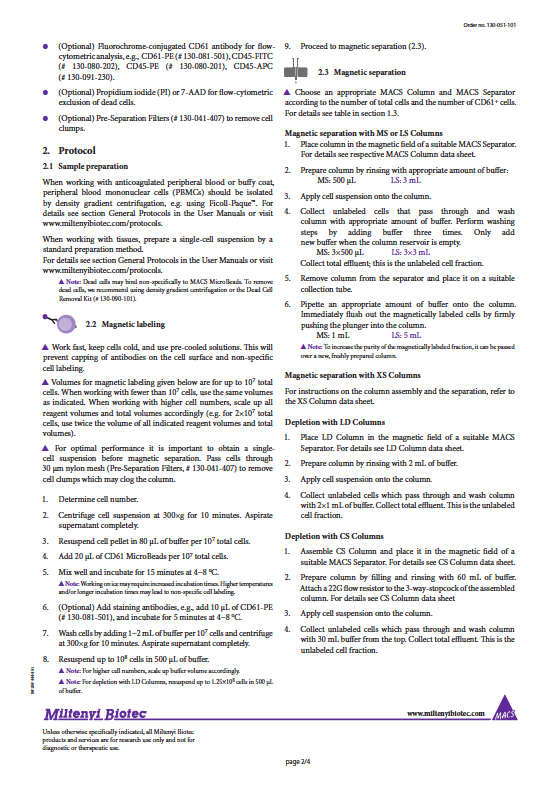


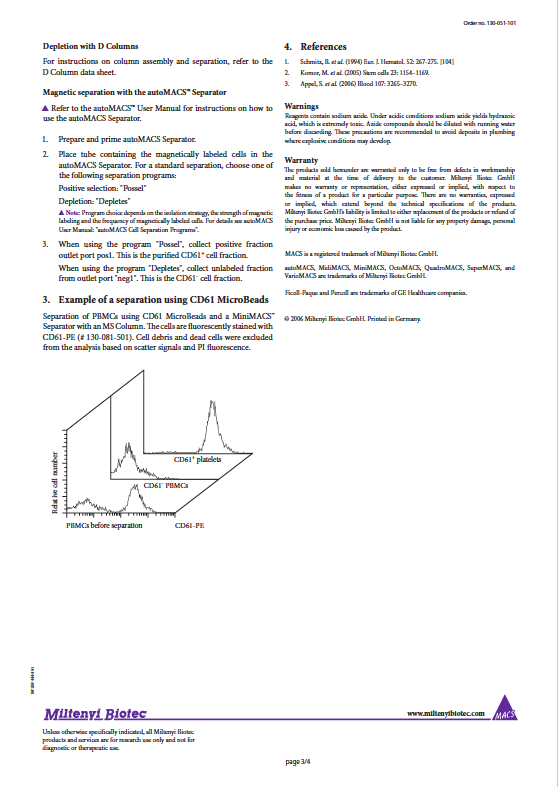


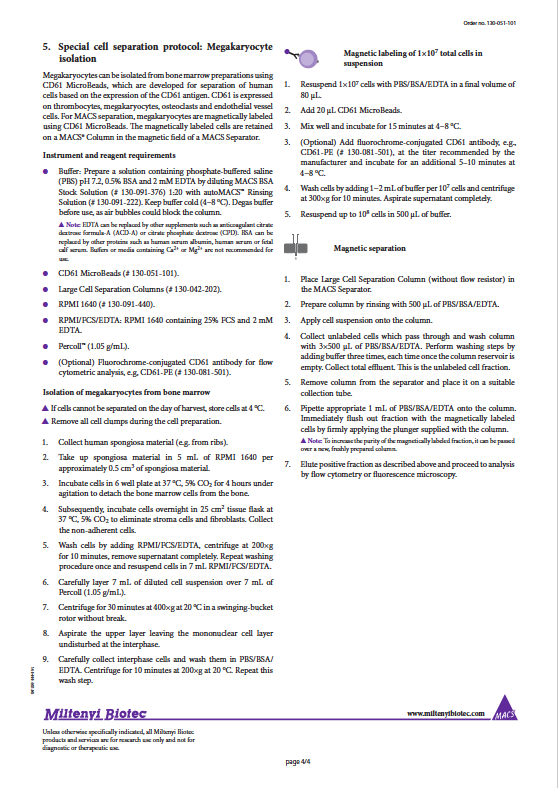


S1 Datafile D Naïve B Cell Isolation Kit II


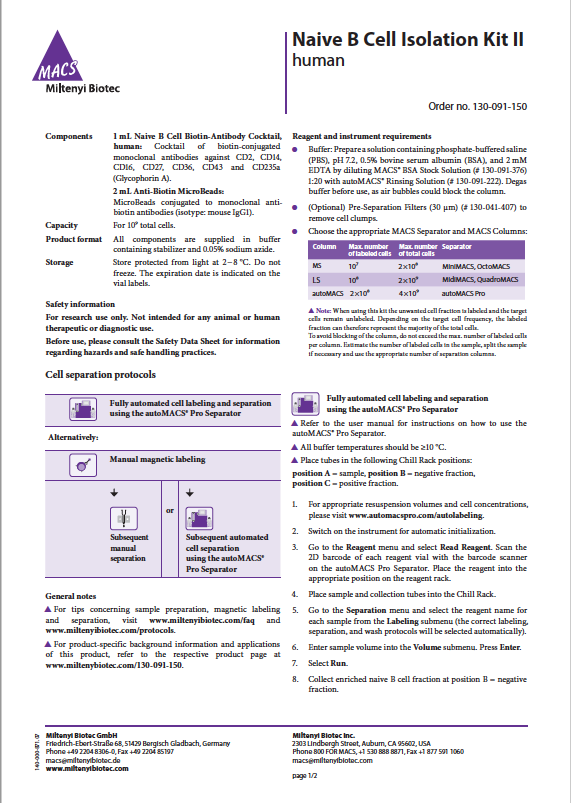


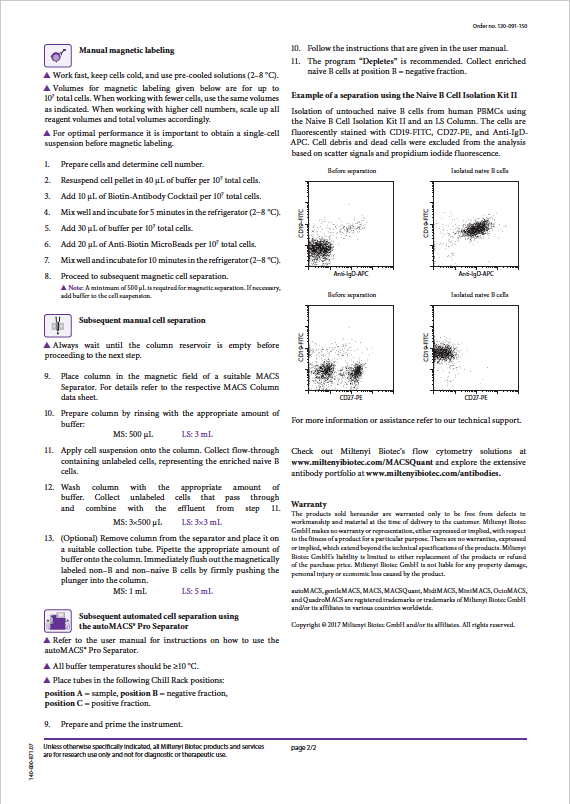


S1 Datafile E CD43 Microbeads


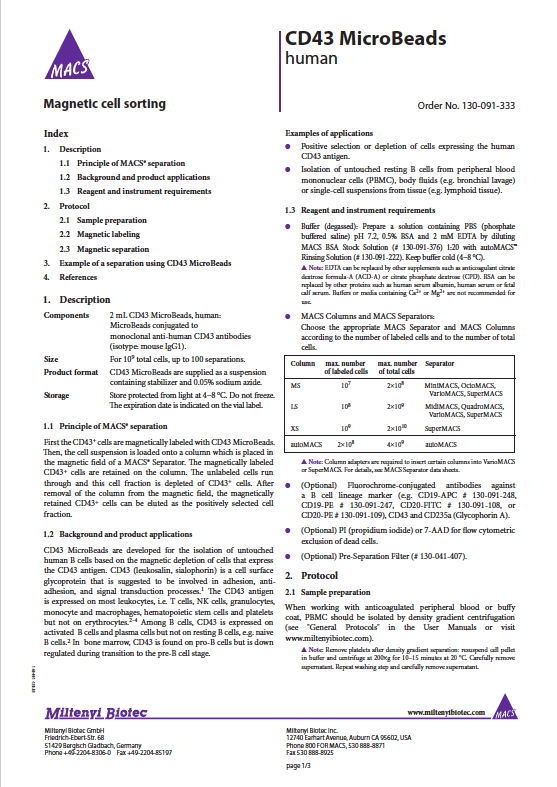


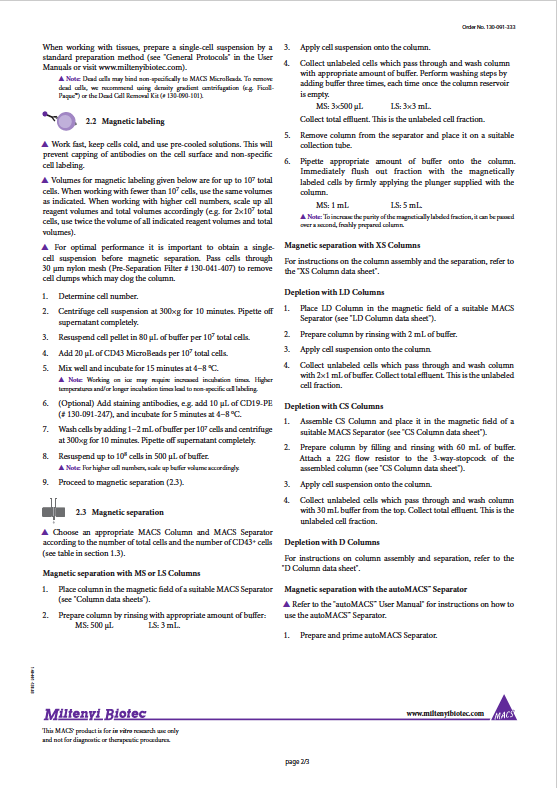


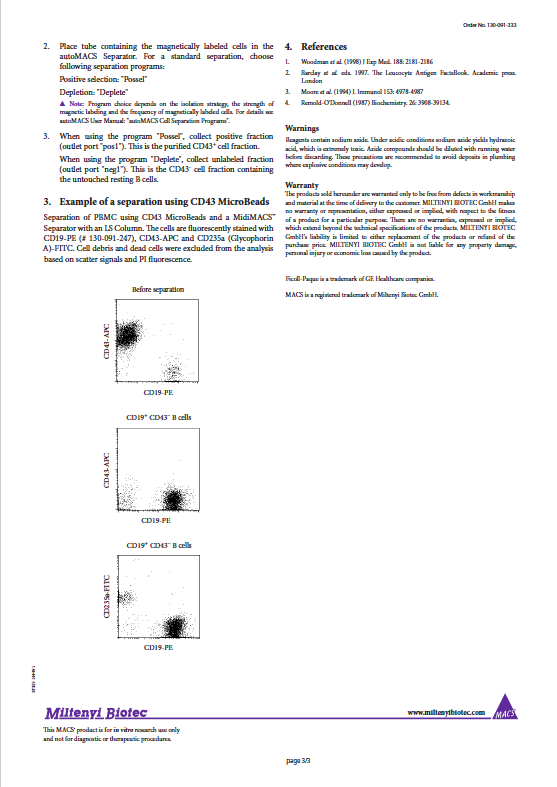


S1 Datafile F CD19 Microbeads


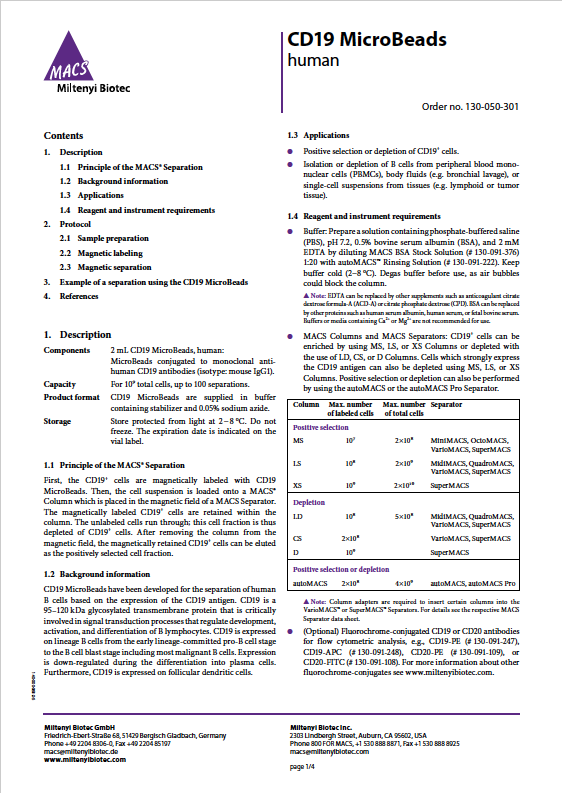


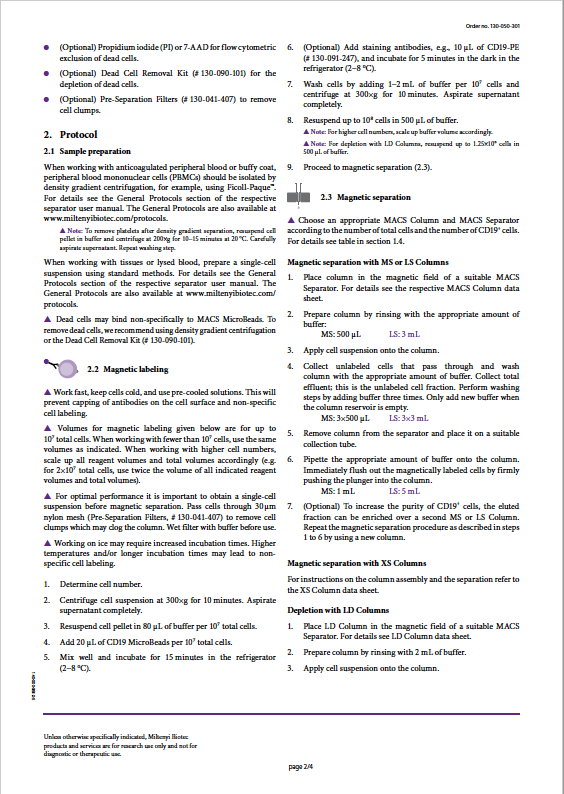


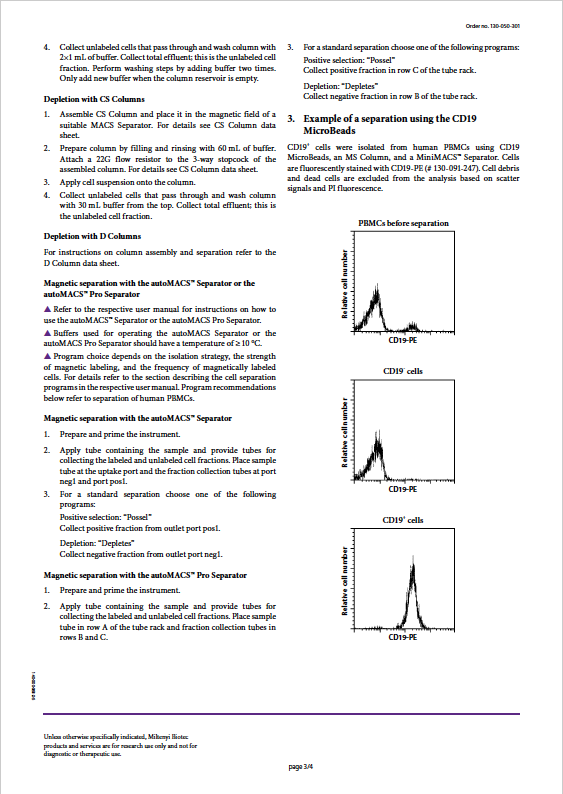


**Supplementary Data**

a) b)

c) d)


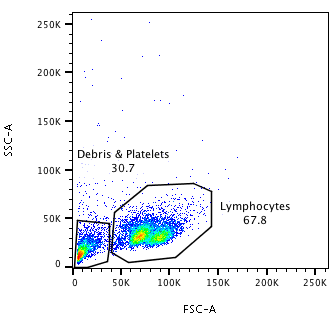

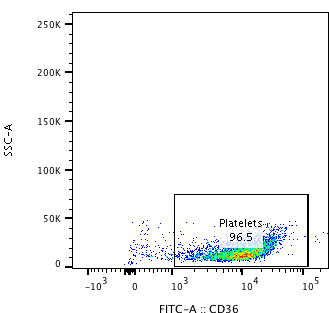

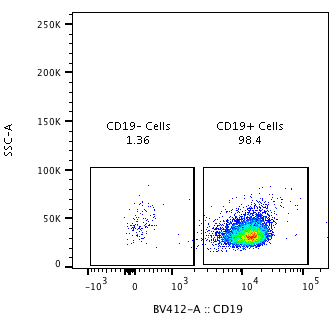

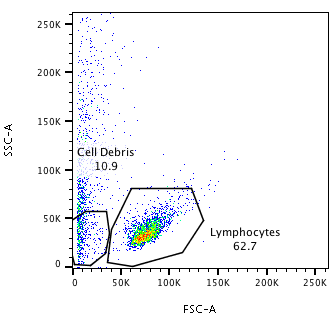

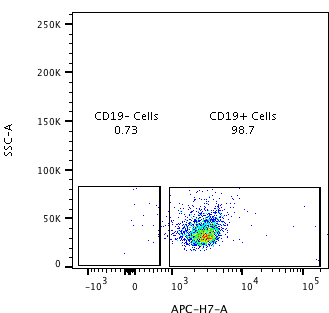

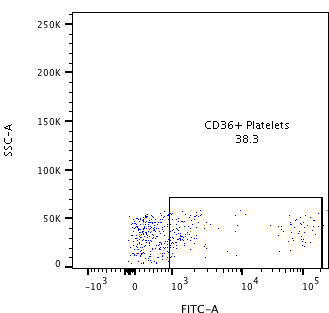

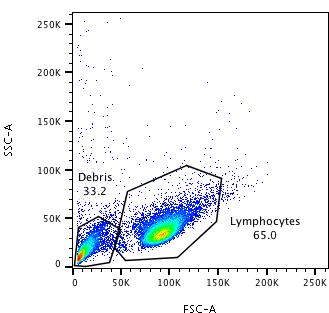

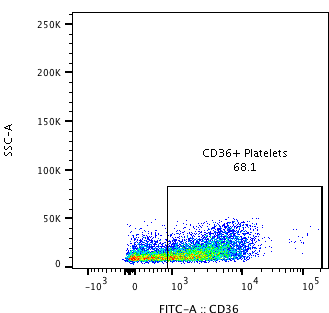

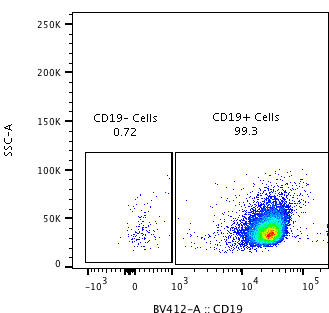

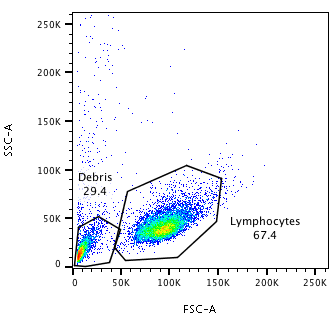

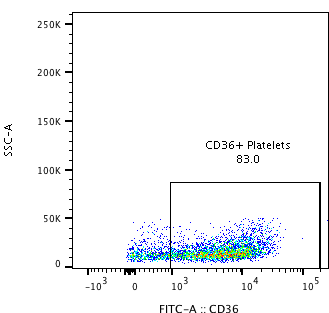

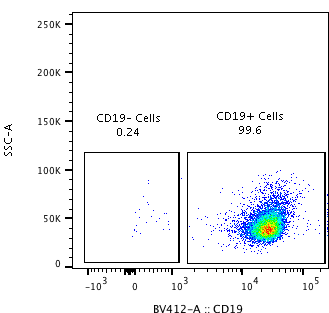


**Figure A**

**S1_Fig A.** **Flow cytometric analysis of isolated B cell sample purity obtained using the B cell isolation kit II.** (a) Using the normal PBMC method (n = 16) (b) Using the altered platelet wash method (n= 4) c) Addition of dead cell removal kit (n = 3) (d) Addition of CD61 platelet removal kit ( n = 3)

**Figure B**

a) b)

c)


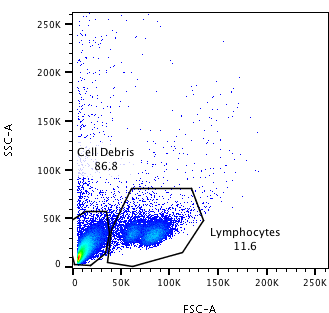

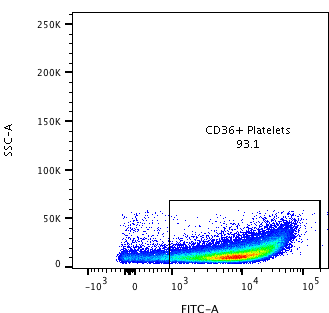

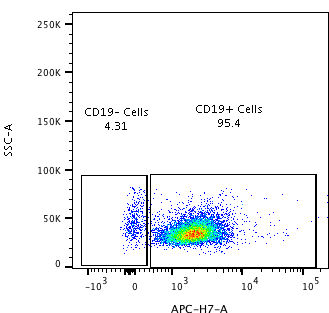

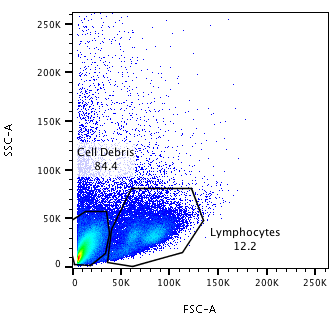

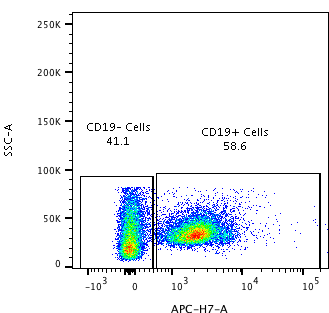

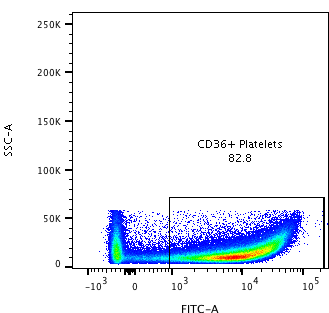

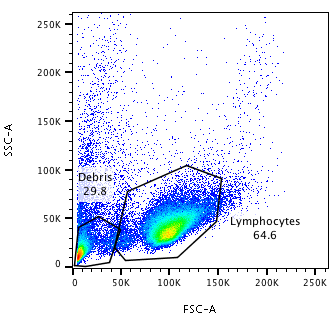

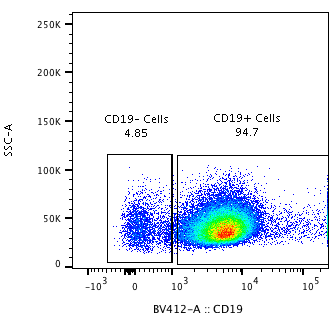


**S1_Fig B.** **Flow cytometric analysis of isolated B cell sample purity obtained using the naive isolation kits**. (a) Naïve B cell Isolation kit (n = 1) (b) CD43 microbeads kit (n = 1) (c) CD19 positive Isolation kit (n = 3). These sample were not stained with CD36 mAb and thus the proportion of platelets that make up the ‘Debris’ population cannot be confirmed.

**Figure C**


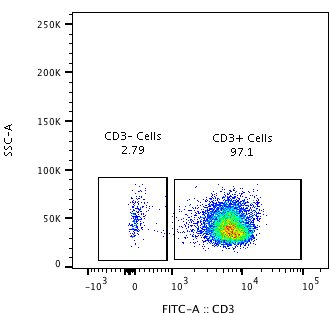

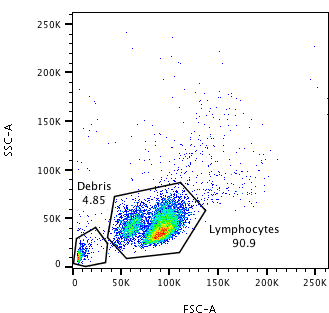


**S1_Fig C.** **Flow cytometric analysis of isolated T cell sample purity obtained using the Pan T cell isolation kit.**Sample purity following negative MACS bead isolation in which platelet/cell debris was successfully removed, as shown in SSC-A vs FSC-A, and a pure T cell population obtained, as shown by CD3+ cells (n = 11)

**S1_Fig D**

**S1_Fig D.** **Flow cytometric analysis of isolated B cell sample purity obtained using the B cell isolation kit II, followed by cell sorting based on FSC and SSC.**a) Sample purity of MACS bead isolated B cell sample b) Sample purity of MACS bead isolated B cell sample followed by two cell sorting steps, resulting in successfully removal of undesirable platelet contamination (n=2)

a) b)


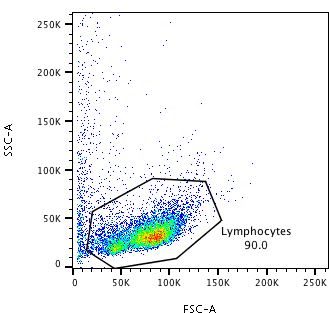

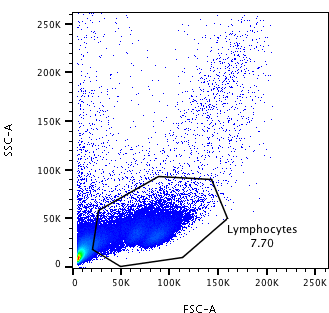

Supplement: S1 File — Bcell Isolation kit II (Datafile A). Dead Cell Removal Kit (Datafile B). CD61 Microbeads (Datafile C). Naïve B cell Isolation Kit II (Datafile D). CD43 Microbeads (Datafile E). CD19 Microbeads (Datafile F). Flow cytometric analysis of isolated B cell sample purity obtained using the B cell isolation kit II. (a) Using the normal PBMC method (n = 16) (b) Using the altered platelet wash method (n = 4) c) Addition of dead cell removal kit (n = 3) (d) Addition of CD61 platelet removal kit (n = 3) (Figure A). Flow cytometric analysis of isolated B cell sample purity obtained using the naive isolation kits. (a) Naïve B cell Isolation kit (n = 1) (b) CD43 microbeads kit (n = 1) (c) CD19 positive Isolation kit (n = 3). These sample were not stained with CD36 mAb and thus the proportion of platelets that make up the ‘Debris’ population cannot be confirmed (Figure B). Flow cytometric analysis of isolated T cell sample purity obtained using the Pan T cell isolation kit. Sample purity following negative MACS bead isolation in which platelet/cell debris was successfully removed, as shown in SSC-A vs FSC-A, and a pure T cell population obtained, as shown by CD3+ cells (n = 11) (Figure C). Flow cytometric analysis of isolated B cell sample purity obtained using the B cell isolation kit II, followed by cell sorting based on FSC and SSC. a) Sample purity of MACS bead isolated B cell sample b) Sample purity of MACS bead isolated B cell sample followed by two cell sorting steps, resulting in successfully removal of undesirable platelet contamination (n = 2) (Figure D). (DOCX) [file pone.0213832.s001.docx]
